# Initial Experiences with the Envoy Acclaim^®^ Fully Implanted Cochlear Implant

**DOI:** 10.3390/jcm12185875

**Published:** 2023-09-10

**Authors:** James R. Dornhoffer, Skye K. Lawlor, Aniket A. Saoji, Colin L. W. Driscoll

**Affiliations:** Department of Otolaryngology—Head and Neck Surgery, Mayo Clinic, Rochester, MN 55905, USA; dornhoffer.james@mayo.edu (J.R.D.); lawlor.skye@mayo.edu (S.K.L.); saoji.aniket@mayo.edu (A.A.S.)

**Keywords:** cochlear implant, fully-implantable, internal device

## Abstract

**Introduction:** Cochlear implantation has become the standard of care for the treatment of moderate-to-profound bilateral sensorineural hearing loss. However, current technologies, all of which rely on an external sound processor, have intrinsic limitations that prevent certain activities and diagnostics, thus hampering full integration into a patient’s lifestyle. The Envoy Medical (White Bear Lake, MN, USA) Acclaim^®^ fully implanted cochlear implant is a new device currently undergoing testing that has been designed to alleviate many of the current constraints by housing all components within the patient, thus allowing for near-constant use in many environments that are not conducive to a traditional cochlear implant. **Methods**: As part of an Early Feasibility Study, three adult implant candidates were implanted with the Acclaim^®^ cochlear implant. Surgical video and photography were taken, and initial observations were recorded. Implantation with the Acclaim^®^ device is largely similar to a traditional cochlear implant, with modifications to allow room for the implanted sensor as well as the implantation of a battery in the subcutaneous tissues of the chest. **Results:** This study demonstrates a step-by-step overview of implanting the Acclaim^®^ and discusses initial insight and experiences with the first three implantations with this new device. **Conclusions:** All three surgeries proceeded without complication, and at activation, all three patients were hearing through their devices. Surgery is more technically challenging compared to a standard cochlear implant, but the skills needed can all be mastered by a dedicated otologic surgeon.

## 1. Introduction

Cochlear implantation has become the standard of care for those patients with moderate-to-profound bilateral sensorineural hearing loss, with demonstrable improvements in both speech recognition and quality of life after implantation [1,2]. This success has prompted the steady broadening of cochlear implant (CI) candidacy criteria, with the number of implant recipients currently approaching 1 million worldwide [3].

Despite these successes, a number of technological limitations are still prevalent. Current devices rely on an external sound processor that transmits the appropriate signal to the internal device and electrode array [4]. While not much more invasive than many large hearing aids, the processor is generally affixed to the skull with a magnet. The pressure from this attachment can cause pain or skin breakdown for some patients [5], and the presence of this magnet can cause difficulty with MRI imaging, which many patients may require [4,6,7]. The external device can also be damaged, lost, or cause discomfort due to its bulk, thereby limiting its use under certain situations, such as strenuous physical activity, water exposure, and while sleeping.

The Envoy Acclaim^®^ attempts to alleviate the limitations of an external CI by making the device fully implanted, with no external components, except a charger and charger-harness that can be used as needed. This is performed with an implanted sensor that articulates with the incus, similar to the Envoy Medical (White Bear Lake, Minnesota, USA) Esteem^®^ implantable hearing aid, and an internal battery mounted in a similar fashion to a pacemaker or nerve stimulator battery pack. There is an external handheld charger that patients use to change device settings and charge the implanted battery. The implanted sensor captures acoustic energy from the movement of the ossicular chain and transmits this information to the cochlear implant that then stimulates the cochlea through a standard electrode array. In this manner, the patients’ ossicular chain and the attached sensor replace the receiver that is generally worn externally while still providing information to the cochlear implant for stimulation.

This study details the initial experiences with the Envoy Acclaim^®^ device and provides a step-by-step view of the surgical procedure, highlighting key similarities, differences, and constraints of the device compared with a traditional CI [2].

## 2. Materials and Methods

This study was undertaken as part of an ongoing Early Feasibility Study for the Envoy Medical Acclaim^®^ fully implanted CI. The study was approved by the Mayo Clinic Internal Review Board (protocol number 20-003924). Any procedures were performed only after full informed consent pursuant to the trial protocol.

### 2.1. Patient Selection

Key inclusion criteria were as follows: ≥18 years of age, good health and absence of significant morbidity, English fluency, post-lingual deafness, pure-tone thresholds indicative of at least a bilateral severe-to-profound sensorineural hearing loss with limited benefit from amplifications (defined by an aided consonant-nucleus-consonant word score of ≤40% in the ear to be implanted and ≤60% in the contralateral ear), and normal middle ear function (as detailed by exam and tympanometry (type A, AS, or AD)).

Key exclusion criteria were as follows: more than 10 years of documented severe-to-profound hearing loss; an air-bone gap greater than 10 dB at two of 500, 1000, 2000 and/or 4000 Hz; prior surgery in the middle ear, inner ear, neck, or interclavicular fossa that is anticipated to prevent proper placement of the device; a cochlear anomaly that might prevent complete electrode insertion; and diagnosed auditory neuropathy or other retrocochlear cause of hearing loss.

### 2.2. Surgical Details

All three surgeries were performed by the senior author (CLWD). Surgeries were performed in a large tertiary medical center in the routine ambulatory operating suite, and patients were discharged on the same day of surgery.

Photography was obtained throughout the procedure using the integrated video system of a Zeiss (Oberkochen, Germany) Kinevo^®^ surgical microscope and separate optical cameras. Photos were taken of all three surgeries, and best representative images were chosen to describe key findings from a surgeon’s point of view.

All intraoperative audiometry was performed by author AAS in the presence of the Envoy Medical team.

## 3. Results: Step-by-Step Surgical Approach

### 3.1. Position and Incision

Following anesthesia induction, intubation, and preparation of cranial nerve (CN) monitoring, the patient is positioned supine with the head turned away from the surgeon. An insert speaker is placed into the ipsilateral ear canal. This will provide a stimulus for testing later in the procedure. The patient is then sterilely prepped and draped.

**Figure 1:**
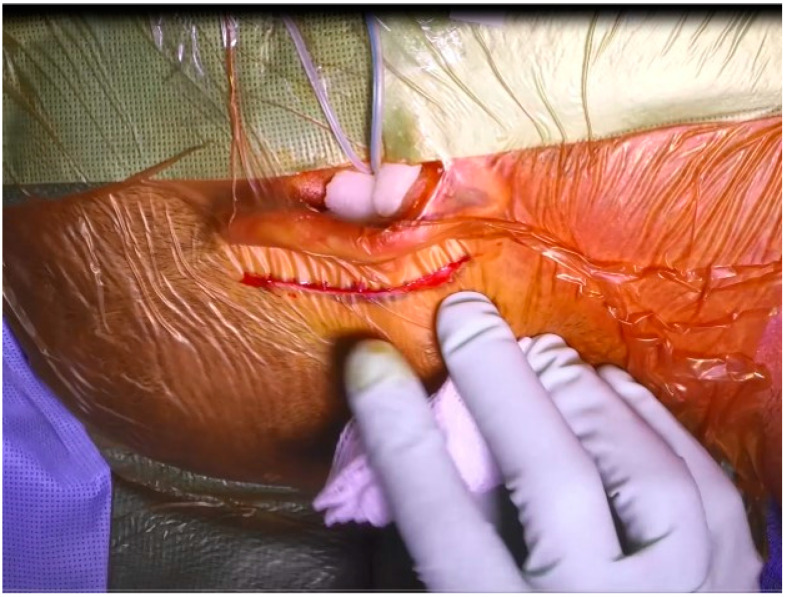
Insert microphone and postauricular incision.

A 4-cm incision is then made 0.5–1 cm posterior to the postauricular crease, Figure 1. Depending on surgeon preferences, this incision can be extended superiorly more than would be used for a traditional implant. This provides exposure to the squamous temporal bone that will be used to support a positioning device used later in the procedure. After the incision is made, soft tissue is harvested to pack the round window, and an anteriorly based flap is elevated, providing adequate exposure to the mastoid.

### 3.2. Mastoidectomy with Facial Recess

**Figure 2:**
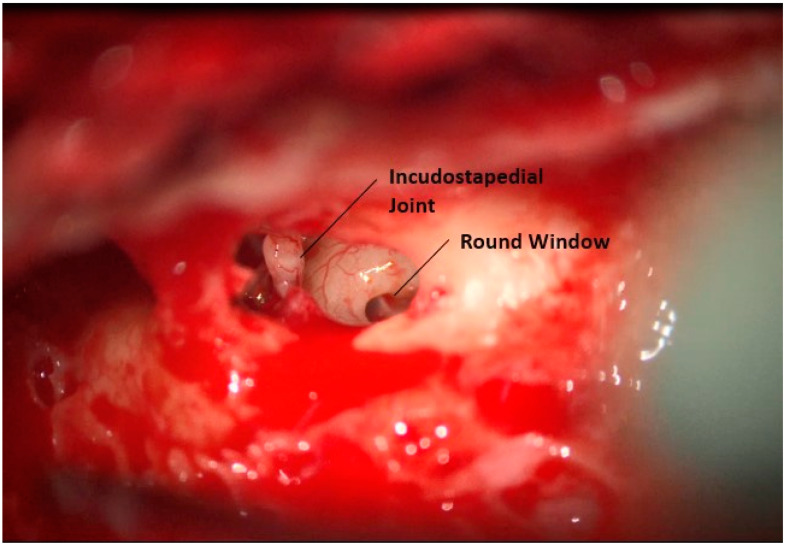
Opening of facial recess.

A mastoidectomy is then completed in a similar fashion as would be used for a traditional CI, and the facial recess is opened, Figure 2. The chorda tympani nerve is preserved. The round window niche is drilled, removing the bony overhang without violating the membrane. Care is taken to leave an incus buttress in place during this dissection to protect the ossicular chain from trauma. Differing from a traditional implant, additional bone posterior to the antrum and at the sinodural angle is removed. This is performed to provide room for the sensor portion of the device.

### 3.3. Posterior Atticotomy

**Figure 3:**
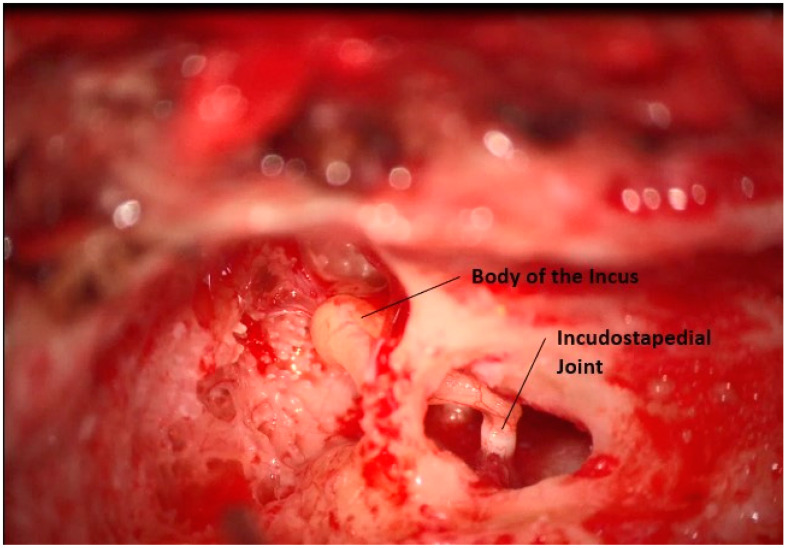
Posterior atticotomy and exposure of the incus short process.

Unlike a traditional CI, the lateral surface of the body of the incus must be widely exposed. To this end, the root of the zygoma is drilled, and a posterior atticotomy is performed until the entire body of the incus is visible in the epitympanum, Figure 3. Care is taken to protect the mucosa on the incus and avoid any injury to the ossicular chain. At this point, all drilling is completed.

### 3.4. Testing Ossicular Mobility

**Figure 4:**
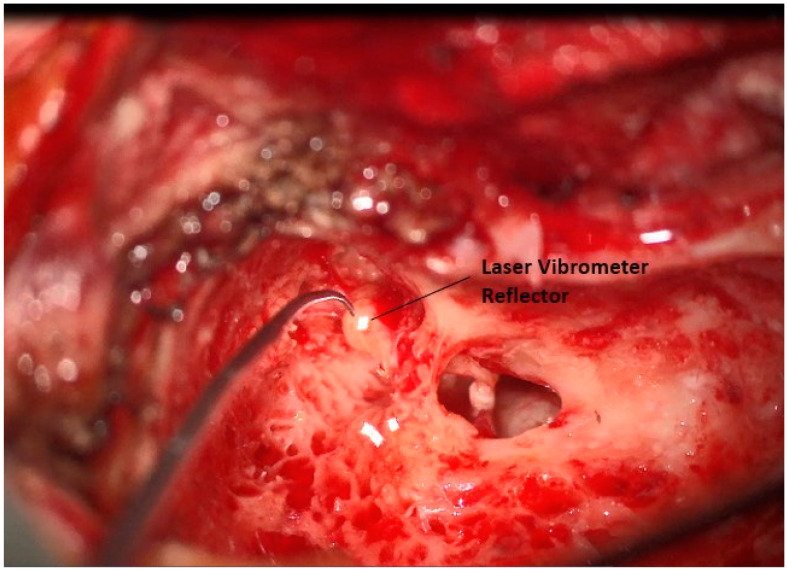
Placement of laser vibrometer reflector on the incus short process.

After wide exposure to the incus body, a small laser vibrometer reflector is placed on the body of the incus, Figure 4. A separate, sterilely draped microscope with a mounted laser vibrometer is then brought into the field and focused on the reflector. Using stimulus from the insert speaker, the vibration of the ossicular chain is tested, and initial calibration measures for the implanted sensor can be taken by our audiology team. After confirmation of good mobility, the reflector is removed.

### 3.5. Placement of Battery in Chest

**Figure 5:**
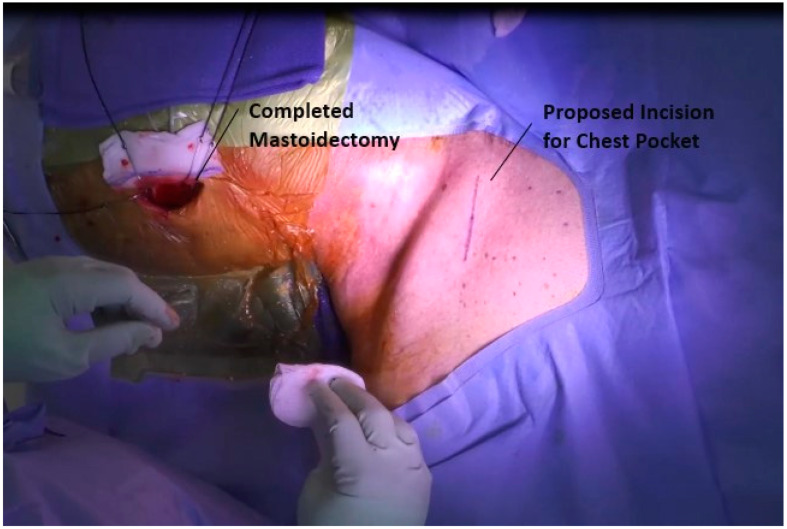
Chest incision.

**Figure 6:**
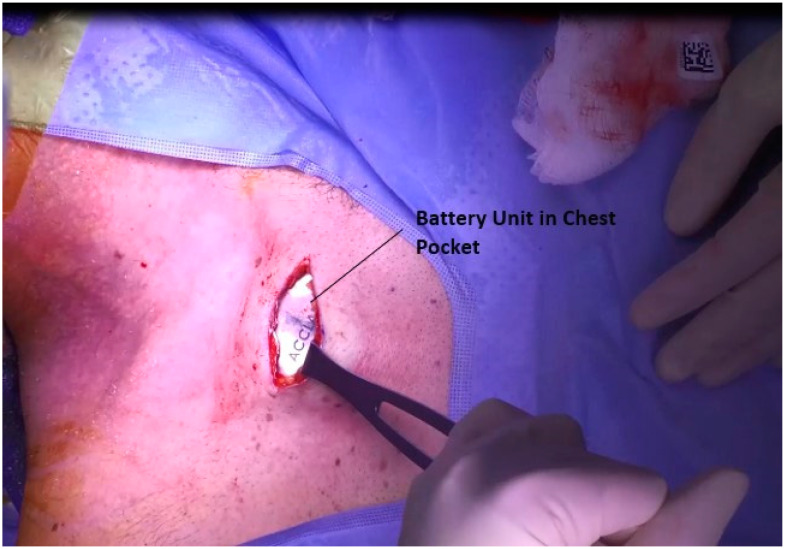
Chest pocket with battery unit in place.

Attention is then turned to the placement of the battery. An ~3 cm incision is made at the midclavicular line, approximately 1 cm inferior to the clavicle, Figure 5. The incision is carried down to the fascia of the pectoralis major, and a pocket is developed inferiorly along the muscle to house the device, Figure 6. This battery also houses the transducer by which the device is accessed for both programming and charging. As such, in a patient with a large body habitus, a more superficial pocket to allow for charging may be necessary.

**Figure 7:**
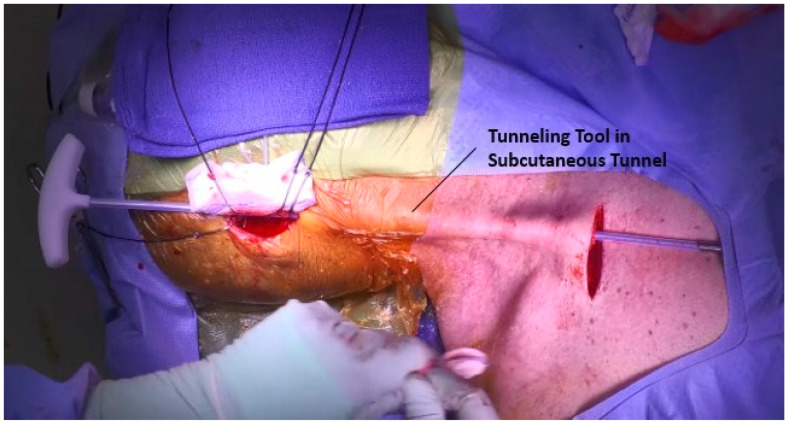
Subcutaneous tunnel from chest to mastoid with tunneling tool in place.

A tunnel is then developed from the mastoid to the chest. This is done using a blunt-tipped tunneling tool and is tunneled in a subcutaneous fashion from the mastoid tip, over the clavicle, and into the chest pocket, Figure 7. The sheath covering the tunneling rod is temporarily retained, through which a lead, which was sized prior to surgery, is passed from the mastoid to the chest and secured into the battery unit. The device is then placed in the pocket with a small amount of vancomycin powder. For efficiency, the battery unit can be placed, and the chest incision closed while the cement anchoring the sensor is drying (see next section).

### 3.6. Placement of the Sensor

**Figure 8:**
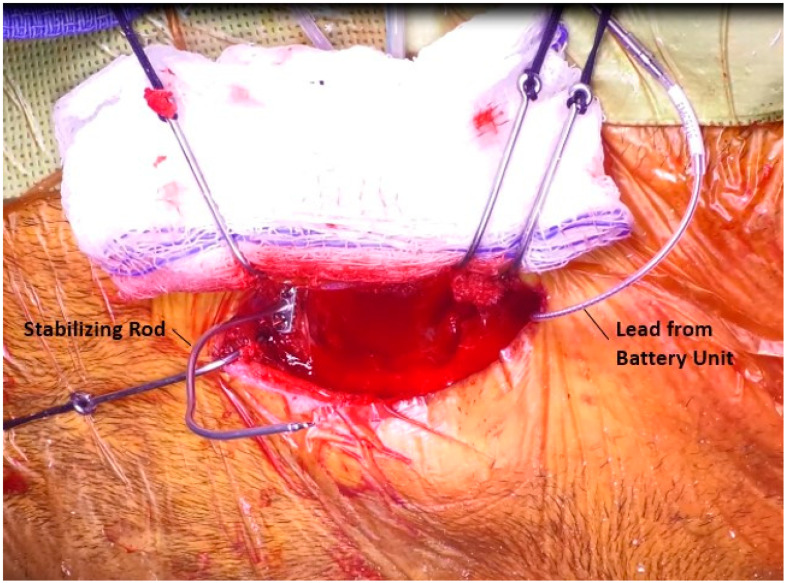
Placement of stabilizing rod on the mastoid cortex.

**Figure 9:**
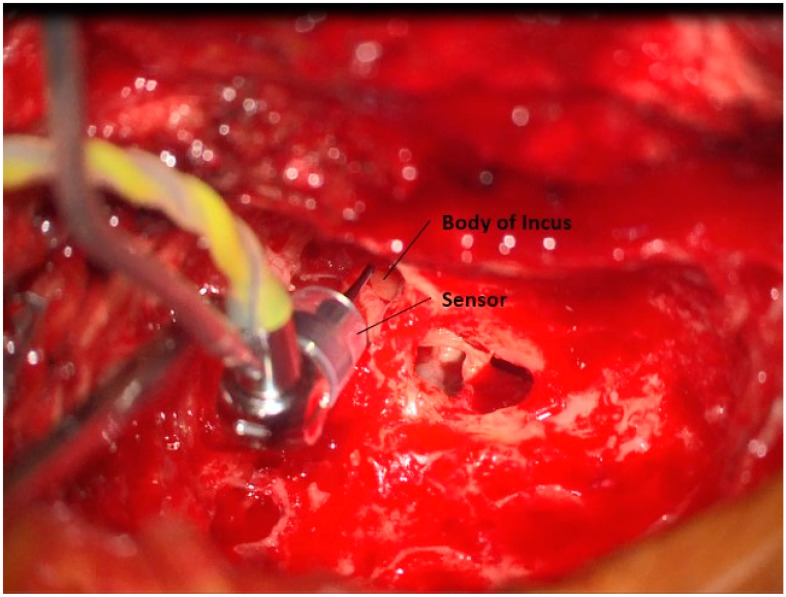
Placement of sensor with distal tip just lateral to the incus.

**Figure 10:**
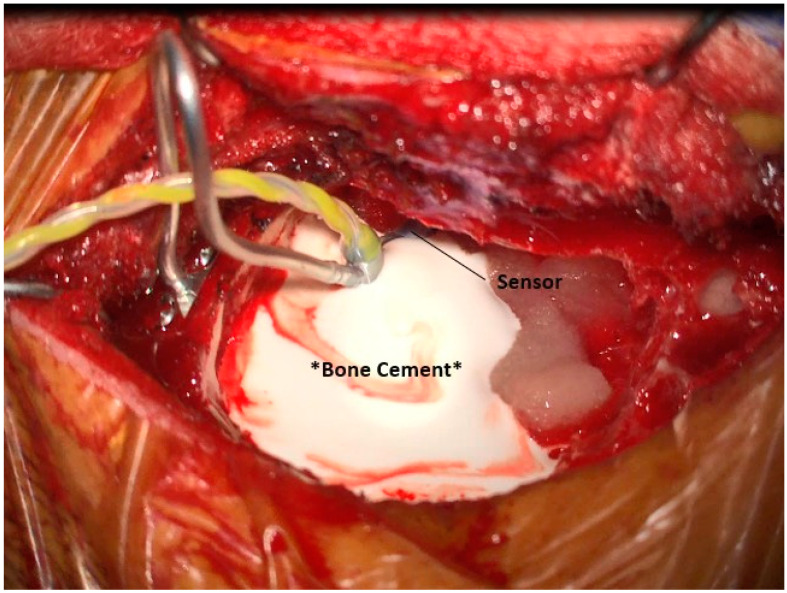
Fixation of sensor with bone cement.

At this point, the mastoid is prepared for placement of the sensor. The sensor site, which was drilled earlier, is irrigated to remove any debris that may impact the placement of the bone cement. A malleable stabilizing rod (Glasscock stabilizer) is then fixed to the squamous temporal bone using self-tapping screws, Figure 8. The sensor is fit onto the rod and is moved into its appropriate location, with the distal tip of the sensor just lateral to the body of the incus, Figure 9. Once a good location is achieved, bone cement is placed around the body of the sensor, taking care to keep the cement away from the anterior, mobile portions of the sensor. Gelfoam is used to guard mobile elements and the facial recess, Figure 10. The stabilizing rod keeps the sensor in place while the cement dries. Multiple applications of cement may be necessary to ensure a solid fit. After the cement is dry, the stabilizing rod is removed.

### 3.7. Creation of the Sensor Neo-Joint

**Figure 11:**
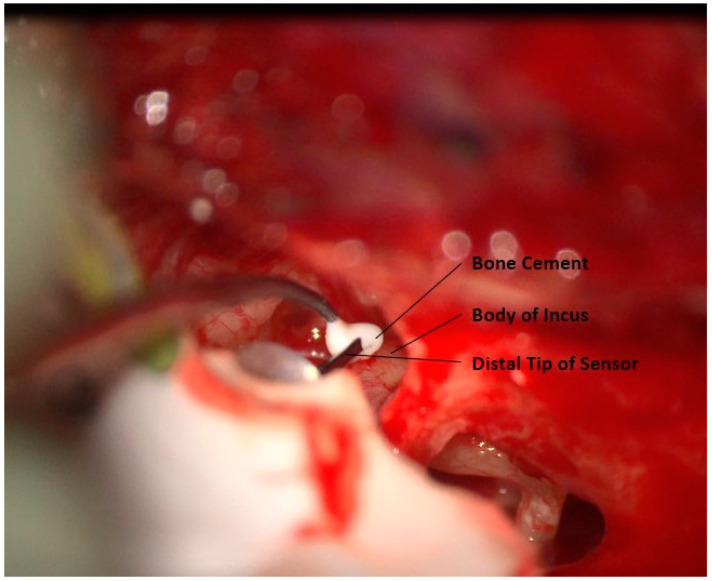
Placement of bone cement to connect the sensor and incus.

**Figure 12:**
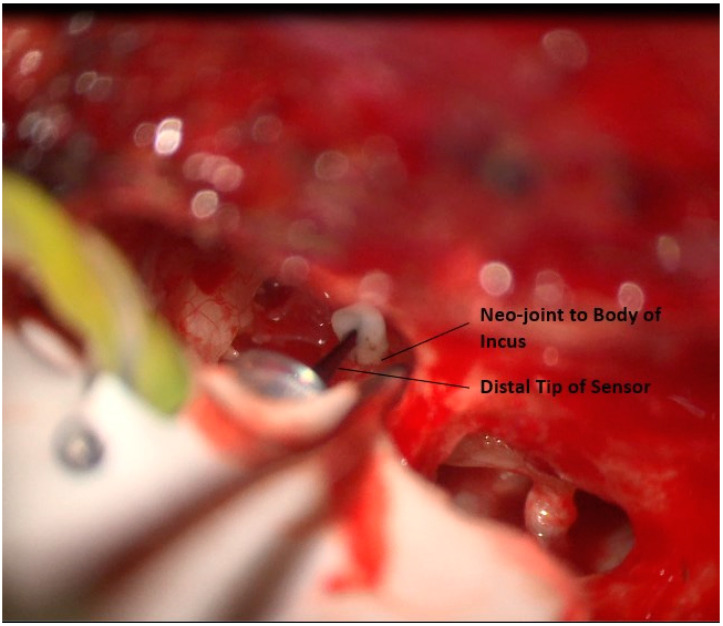
Mobilization of bone cement on incus to create a neo-joint with the sensor.

A neo-joint is created between the distal tip of the sensor and the body of the incus. A small drop of bone cement is placed at the sensor tip. The bone cement contacts the tip and body of the incus, Figure 11. This is allowed to dry. After the cement is dry, the cement is carefully mobilized from the body of the incus while keeping fixed to the sensor, creating a neo-joint between the sensor and the incus. This step is facilitated by maintaining the mucosa on the incus, creating a natural plan for separation Figure 12. In this manner, close contact is maintained between the incus and the sensor without creating a fixed ossicular chain that could dampen sound. The sensor is then checked by application of stimulus using the ear canal insert speaker.

### 3.8. Placement and Connection of Device

**Figure 13:**
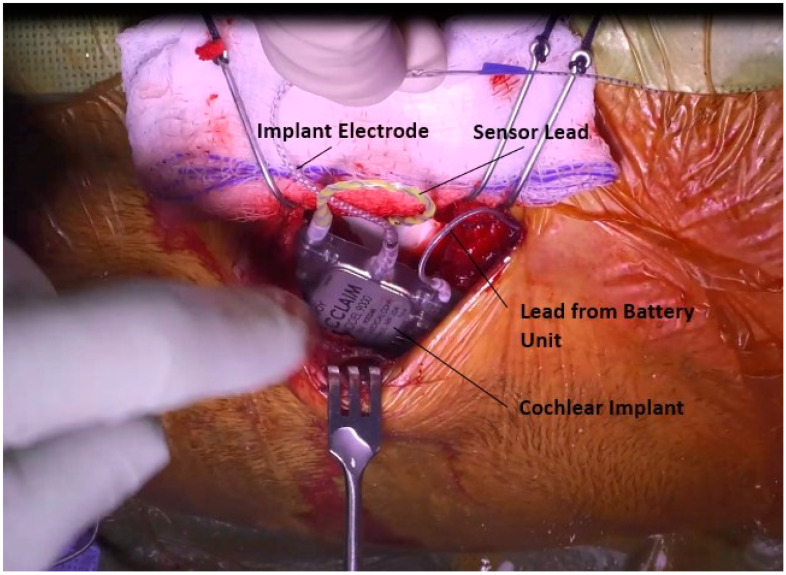
Connection of sensor and battery unit to the processor and placement of processor in a tight subperiosteal pocket.

At this point, the CI itself is placed. A subperiosteal pocket is elevated in the standard fashion. Per surgeon preference, a well or trench can be drilled to accommodate the device or various leads. The leads from the sensor and battery unit are both connected to the device, and it is placed into the subperiosteal pocket, Figure 13.

**Figure 14:**
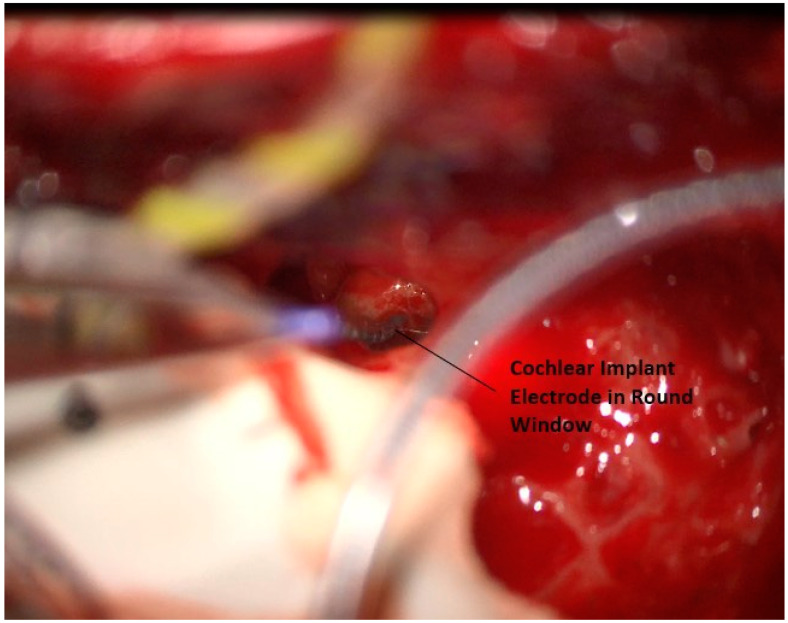
Placement of electrode through round window.

Any remaining round window niche that obstructs the surgeon’s view can be carefully drilled away, and the round window is opened. The electrode is then inserted (Figure 14). The current electrode design is a lateral wall array with 16 electrodes and is placed in the same fashion as similar devices from other companies. After a full-length insertion is completed, the round window is packed with muscle. At this point, both surgical sites are closed with absorbable stitches. Testing is then performed to confirm communication with the device, normal impedances, neural responses, and that a stapedial reflex is consistently elicited. An intraoperative X-ray is obtained to confirm the appropriate positioning of the electrode array. In all three patients, intraoperative testing was normal.

## 4. Discussion

By using an implanted sensor linked to the ossicular chain and an implantable power supply, the Envoy Acclaim^®^ CI aims to obviate the need for any external components. Here, we have demonstrated our initial surgical experiences with this device. We note that the placement of the Acclaim^®^ device is similar to a traditional CI, with changes to accommodate the new technologies. As such, one would anticipate that an experienced and dedicated otologic surgeon would be able to master the placement of this device in select patients. However, we must consider the key differences and the implications they may have, particularly on patient selection.

First is the placement of the sensor onto the incus. For this sensor to be appropriately placed, a patient must have an intact ossicular chain and a well-aerated middle ear and mastoid. Without both of these, the sensor cannot be placed in the appropriate position, and/or the ossicular chain would not transmit adequate sound to the sensor. As such, patients with varying degrees of chronic ear disease may not benefit from this device. Additionally, patients with a low-hanging tegmen, even in the absence of chronic ear disease, may not be a candidate for this device, as access to the epitympanum may not be feasible.

The placement of the power source also bears some consideration. Patients must have a safe region of soft tissue from the mastoid to the chest wall in which to tunnel the leads. Patients with prior surgery or another anomaly in the neck may prohibit placement of the power source. And even in those patients with normal anatomy, the surgeon must still consider body habitus. In patients with a large body habitus, placing the device closer to the skin rather than on the muscle may be necessary to ensure that the device controller and charging coil can reliably connect. At the other extreme, care must be taken with a patient with a very small body habitus so as not to place the device in a prominent area to avoid discomfort and prevent skin breakdown. Fortunately, all patients included in this trial underwent placement with no injury or unforeseen surgical obstacle. Surgical principles learned through the placement of cardiac devices over many decades can be applied to this device.

Ultimately, successful utilization of the Acclaim^®^ device may portend many immediate and long-term benefits. In the immediate interim, the Acclaim^®^ allows patients to use their device in circumstances that would prohibit the use of a traditional CI, such as water exposure or strenuous activities, or in situations that would be inconvenient or uncomfortable, such as while sleeping or wearing headwear. While it is only conjecture at this point, one may presume that increased breadth and comfort of use may improve quality-of-life for many recipients, particularly those who feel their disability prevents them from taking part in certain activities. The fully implanted nature of the Acclaim^®^ also allows for constant or near constant use, which has been shown by both Schvartz-Leyzac et al. [8] and Holder et al. [9], using data logging to be positively correlated with speech recognition outcomes.

While initial surgical experiences have been positive, the overall use of this novel device is a developing process. Several important technical challenges have been identified, solutions generated, and plans for deployment to the existing patients are being created. Given that each patient has a unique sound receiver in the form of their own ossicular chain/sensor complex, programming of this device is complex, requiring careful management of variable input and output levels, and is currently an ongoing process. As such, we cannot speak to the nature or ease of programming of this new device, nor can we speak in depth to speech outcomes at this time. We must also consider the integrity of the sensor device and battery over time, but such information is beyond the scope of this early trial. However, given initial impressions and successful implantations thus far, the Acclaim^®^ device represents an important advancement in our approach to auditory rehabilitation in patients with sensorineural hearing loss.

## Data Availability

All data on the surgical placement of this device is detailed herein. No new data is available for review elsewhere.
